# *SLC35B4*, an Inhibitor of Gluconeogenesis, Responds to Glucose Stimulation and Downregulates Hsp60 among Other Proteins in HepG2 Liver Cell Lines

**DOI:** 10.3390/molecules23061350

**Published:** 2018-06-04

**Authors:** Brigitte Wex, Rémi M. Safi, Gregory Antonios, Perla Z. Zgheib, Dania B. Awad, Firas H. Kobeissy, Rami A. Mahfouz, Marwan M. El-Sabban, Soha N. Yazbek

**Affiliations:** 1 Department of Natural Sciences, School of Arts and Sciences, Lebanese American University, P.O. Box 36, Byblos, Lebanon; gregory.antonios@gmail.com (G.A.); daniabawad@gmail.com (D.B.A.); 2 Department of Anatomy, Cell Biology and Physiological Sciences, Faculty of Medicine, American University of Beirut, Beirut 1107 2020, Lebanon; rs176@aub.edu.lb (R.M.S.); me00@aub.edu.lb (M.M.E.-S.); 3 Medical Laboratory Sciences Program, Faculty of Health Sciences, American University of Beirut, Van Dyck-Room 323, Riad El Solh 1107 2020, Beirut 1107 2020, Lebanon; Perla.zgheib@gmail.com; 4 Department of Biochemistry and Molecular Genetics, Faculty of Medicine, American University of Beirut, Beirut 1107 2020, Lebanon; Firasko@gmail.com; 5 Department of Pathology and Lab Medicine, Faculty of Medicine, American University of Beirut, Beirut 1107 2020, Lebanon; rm11@aub.edu.lb

**Keywords:** solute receptor, gluconeogenesis, downstream effect, HSP60, mass spectrometry, protein analysis, diabetes

## Abstract

*SLC35B4*, solute receptor for UDP-*N*-acetylglucosamine and UDP-xylose, is associated with diabetes and predisposing conditions. This study investigated the localization of *SLC35B4* and compared the differential expression between a knockdown of *SLC35B4* and controls in HepG2. Responsiveness to glucose, expression, and localization were assayed using Western blot and immunostaining. Localization was confirmed using a proximity ligation assay. Two-dimensional (2D) gel electrophoresis and MALDI-TOF were used to identify differentially expressed proteins and pathway analysis was performed. *SLC35B4* was increased by 60% upon glucose stimulation and localized in Golgi apparatus and endoplasmic reticulum. Presence of *SLC35B4* in the Golgi apparatus suggests its involvement in the biosynthesis of glycoconjugate proteins. Four proteins were markedly under-expressed (Hsp60, HspA8, TUBA1A, and ENO1) and linked to the pathogenesis of diabetes or post-translationally modified by *O*-GlcNAc. Glucose levels activate *SLC35B4* expression. This triggers a downstream effect via Hsp60 and other proteins. We hypothesize that the downstream effect on the proteins is mediated via altering the glycosylation pattern inside liver cells. The downstream cascade ultimately alters the ability of cultured liver cells to inhibit endogenous glucose production, and this could play a role in the association of the above-listed genes with the pathogenesis of diabetes.

## 1. Introduction

Diabetes is one of the most common endocrine disorders worldwide, imposing tremendous health and economic burdens [1]. In 2014, worldwide diabetes health expenditure ranged between 612–1099 billion U.S. dollars [2]. Out of all diabetes cases, Type II diabetes (T2D) accounts for around 90% and its prevalence has dramatically increased [3]. Insulin resistance (IR) remains the most important risk factor in T2D development [4,5]. The resulting hyperglycemia is currently controlled by lifestyle modifications or medications, such as sulfonylureas or metformin, which dramatically reduce morbidity. However, these therapies frequently fail to achieve optimal glycemic levels, which is particularly important to minimize the risk of microvascular and other complications [6]. There is overwhelming evidence in the literature that susceptibility to T2D is highly heritable, ranging from 20 to 80% [7]. Despite considerable progress with gene identification, most susceptibility genes continue to elude discovery. The cumulative contribution of the identified genetic variants accounts for only 10% of the total heritability [7,8]. Hence, it is important to understand more about the genetic control of T2D and investigate new genes involved in glycemic control. This would lead to the development of better interventions and treatments as well as to the ability to identify high-risk individuals for monitoring and prevention.

One such novel gene, recently implicated in glycemic control, is *SLC35B4*. This gene is a human solute receptor gene located on chromosome 7q33 for which two splice variants, a longer version (encoding a protein of 331 amino acids) [9,10] and a shorter version (encoding a protein of 231 amino acids) [10], have been reported. *SLC35B4* mRNA can be detected in most tissues [11]. *SLC35B4* encodes a protein that exhibits a dual activity in transporting UDP-*N*-acetylglucosamine (UDP-GlcNAc) and UDP-xylose (UDP-xyl). It is the only identified UDP-xyl transporter so far. UDP-xyl is required for glycosaminoglycan biosynthesis on the core protein of proteoglycan sugar chains [12], whereas UDP-GlcNAc is the major end product of the hexosamine biosynthesis pathway [13]. UDP-GlcNAc can be considered a sensor for the nutritional state of the cell, as it integrates glucose, glutamine, fatty acids, uridine, and ATP metabolism. It is a substrate for *O*-GlcNAc transferase (OGT), which catalyzes the O-linked *N*-acetylglucosaminylation (*O*-GlcNAcylation) occurring in the cytoplasm. The *O*-GlcNAcylation is a reversible post-translational modification (PTM) that controls protein subcellular localization, stability, or activity according to the nutritional environment. It corresponds to the addition of a sugar moiety to serine/threonine residues [14].

Although it is expressed in most tissues, the cellular localization of *SLC35B4* is not clear. It depends on the cellular model used and the spliced variant of the transporter. Masczak-Seneczko et al. showed that both stably overexpressed *SLC35B4* splice variants co-localized in MDCK wild-type and MDCK-RCAr mutant cells with the endoplasmic reticulum (ER) marker only [15]. However, data obtained previously demonstrated that *SLC35B4* (a longer splice variant) is localized to the Golgi apparatus of CHO cells [9]. Although not definitively resolved, function of *SLC35B4* is thought to mainly take place in the Golgi, and UDP-xyl is most likely generated from UDP-GlcA in the ER.

*SLC35B4* has been linked to several metabolic disorders and is involved in obesity-induced T2D. A single nucleotide polymorphism (SNP) in the human *SLC35B4* gene was associated with variation in body mass index (BMI), metabolic syndrome, fasting glucose, pro-insulin levels, and fat stores [16,17]. An increase in *SLC35B4* expression was observed in subcutaneous adipose tissue of obese humans [18].

Genetic screening in mouse models for quantitative trait loci (QTLs) showed that the alteration of hepatic gene expression of *SLC35B4* correlates in vivo with insulin resistance and gluconeogenesis. In vitro experiments conducted on liver-derived tissue culture cells also revealed that altered *SLC35B4* mRNA levels are associated with gluconeogenesis, mimicking the in vivo model [19]. An in vivo mouse model provided the first evidence that relative improvement in the ability to shut down de novo gluconeogenesis and enhance hepatic sensitivity to insulin was associated with hepatic expression of *SLC35B4*. Furthermore, the study showed that *SLC35B4* knockdown in human and mouse liver culture cells, maintained under different glucose conditions, altered glucose production but not glucose uptake in response to nutrient stimulation. The tissue culture assay proved that *SLC35B4* is able to control hepatic glucose production in vitro [19].

We hypothesize that *SLC35B4* may alter the bioavailability of its cargo nucleotide sugars for PTM on cellular proteins. More than 600 proteins, including the insulin receptor, IRS1, NOTCH, AKT, and AMPK are modified with the addition of a UDP-xyl or UDP-GlcNAc moiety; accordingly, any of these may contribute to the pleiotropic presentation of diabetes and predisposing conditions. Predictably, *SLC35B4* reduction would alter liver protein profile; thus, identification of the receptor’s molecular mechanisms may focus on the whole protein composition in cells. Tools such as two-dimensional polyacrylamide gel electrophoresis (2-DE) and matrix assisted laser desorption/ionization time-off light mass spectrometry (MALDI-TOF-MS) enable the study of disease-associated proteomics. These tools are proving to be effective in decoding the molecular basis of diseases, including diabetes mellitus [20]. This powerful experimental approach allows a systematic and comparative analysis of proteomic changes by combining protein separation, differential expression comparison, and mass spectrometric protein identification.

In this study, we aimed to investigate *SLC35B4*’s role in the control of hepatic glucose production, an important mechanism for the response to insulin and for the alteration of glucose levels. We assayed localization, protein expression, and the deferential downstream proteome expression profile of *SLC35B4* and attempted to identify its subsequent effectors and perform a full pathway analysis.

## 2. Results

### 2.1. SLC35B4 Protein Is Markedly Upregulated in HepG2 Cells in Response to Glucose Stimulation

**Figure 1:**
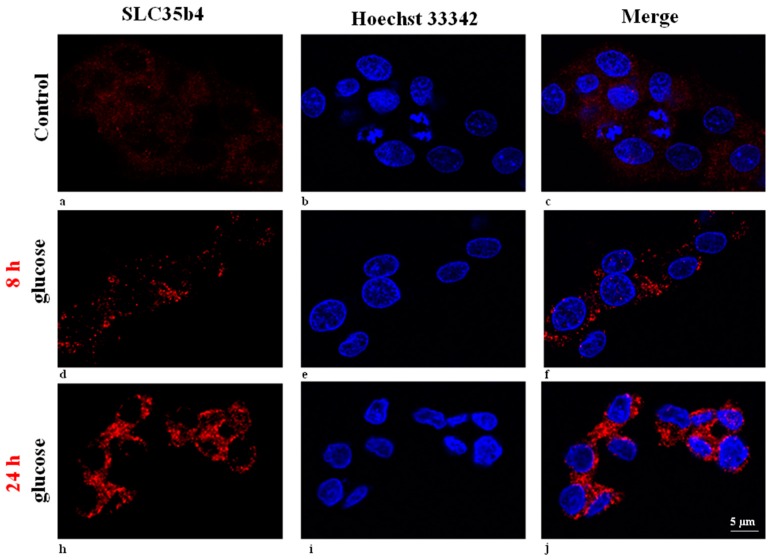
Immunostaining for *SLC35B4* in HepG2 cells. Cells were incubated for 8 h or 24 h in the presence of 10 mM glucose. As a control, immunostaining was performed on untreated cells by omitting the primary antibody. Immunostaining was detected using Alexa fluor555. Cell nuclei were counterstained with Hoechst 33342 dye. Scale bar, 5 μm.

To begin understanding the mechanism by which *SLC35B4* responds to nutrients, we assayed the effect of glucose on the innate expression of *SLC35B4* in liver cells. *SLC35B4* responsiveness to glucose was measured and an immunostaining assay was performed after 8 h and 24 h exposure to 10 mM of glucose (Figure 1). Results showed that *SLC35B4* expression was induced upon glucose stimulation when compared to control non-treated cells. The variation of *SLC35B4* expression occurred in as early as 8 h but the effect was more pronounced at 24 h post-glucose stimulation.

**Figure 2:**
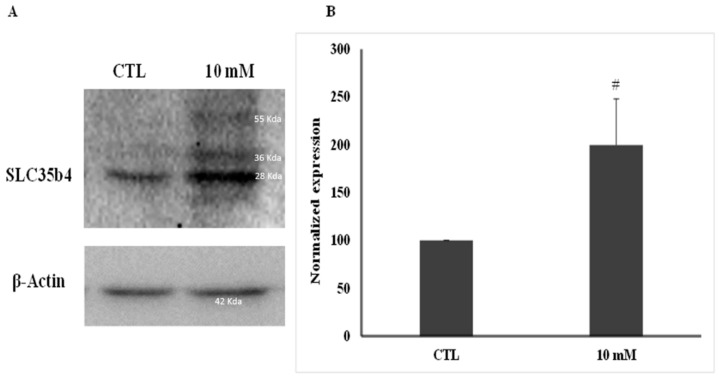
*SLC35B4* protein level upon glucose stimulation. (**A**) *SLC35B4* protein expression by Western blot analysis in HepG2 cells treated with 10 mM glucose for 24 h. *β*-Actin was used as an internal control. The above blot has been cropped. The full length blots are available in the supplementary material (**B**) The quantification of *SLC35B4* levels is represented as percent increase or decrease compared to control levels and was evaluated in four independent experiments. The results were presented as the mean ± standard error of the mean (SEM). # corresponds to *p* < 0.005 versus the control group**.** Control (CTL).

To quantify the upregulation of *SLC35B4* expression in response to glucose, Western blot analysis was performed after 24 h of glucose exposure in HepG2 cells (Figure 2). Results revealed that the *SLC35B4* protein was markedly increased up to 60% upon exposure to high levels of glucose when compared to the control.

### 2.2. SLC35B4 Protein Expression Localizes Mainly to the Golgi Apparatus and the Endoplasmic Reticulum in HepG2 Cells, and Is Abundantly Present in Primary Liver Cells

**Figure 3:**
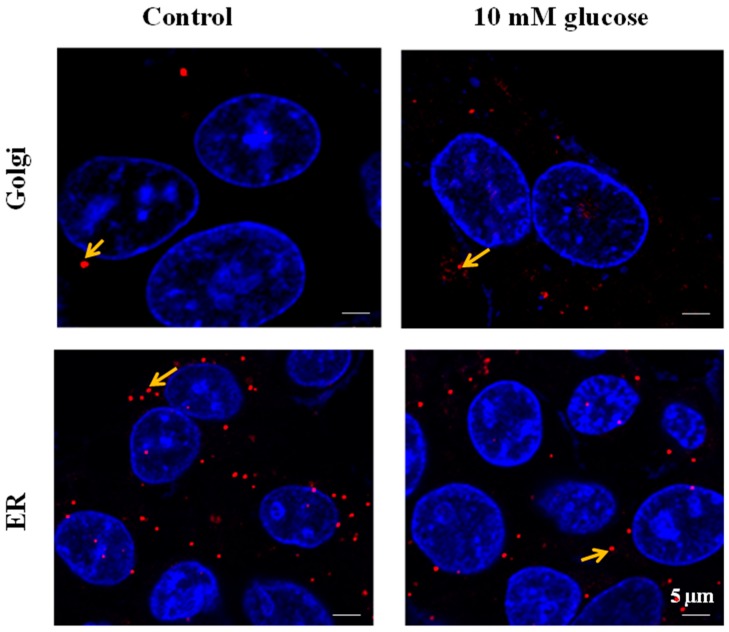
*SLC35B4* interactions with Golgi and endoplasmic reticulum (ER) markers. *SLC35B4* interactions with GM130 (Golgi) and GRP78Bip (ER) detected by duo-link in control/glucose-treated (24 h) HepG2 cells. Arrows point out duo-link dots. The dots are representative of the close proximity of the two proteins of interest. They are indicative of interaction signals between *SLC35B4* protein and Golgi or ER compartments. Nuclei were stained with Hoechst 33342 dye (blue).

*SLC35B4* subcellular localization was assessed using the proximity ligation assay (PLA) technique (Duo-link) for detection of protein–protein interaction in HepG2 cells. Co-localization of *SLC35B4* and GM130 or GRP78 in HepG2 cells was seen using pairs of specific primary antibodies directed against each of the two target proteins (Figure 3). Specific signals were observed in the Golgi and in the ER apparatus as shown by a series of confocal images. Since the PLA will only detect proteins in close proximity to each other, the presence of a signal confirms that *SLC35B4* is localized in Golgi and in ER in both control and treated conditions.

**Figure 4:**
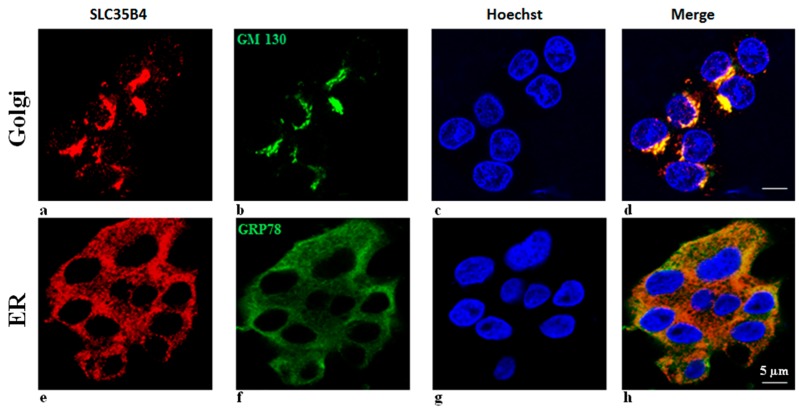
Subcellular localization of *SLC35B4* transporter in HepG2 cells by indirect immunofluorescence. HepG2 cells were cultured and incubated with antibodies as described in Methods. (**a**,**e**) Reactivity with *SLC35B4*-specific antibodies (red), (**b**) reactivity with Golgi marker (GM130) antibodies (green), (**f**) reactivity with ER marker (GRP78) antibodies (green), (**c**,**g**) Cell nuclei were counterstained with Hoechst 33342 dye. (**d**) Overlay of a, b, and c (**h**) overlay of e, f, and g. The intensity of the signal was increased to clearly show localization to subcellular compartments. (**a**,**e**) Reactivity with the *SLC35B4*-specific antibodies signal pattern based on different imaging planes selected for signal intensity of “b” and f”, respectively.

In attempt to identify the cytoplasmic compartment harboring *SLC35B4* in the absence of glucose stimulation, double immunofluorescence studies were performed using: (a) the monoclonal anti-GM130 antibody directed against the Golgi apparatus; and (b) an anti–GRP78 antibody recognizing ER. In HepG2 cells, *SLC35B4* and GM-130 (Figure 4) staining patterns were coincident. This staining pattern demonstrated that *SLC35B4* is a Golgi-resident protein. Nonetheless, the localization of *SLC35B4* partially overlaps with that of GRP78, which specifically localizes to the ER. These two markers of the Golgi complex and ER showed a clear co-localization with *SLC35B4* staining, suggesting a widespread distribution of *SLC35B4* in these two compartments.

**Figure 5:**
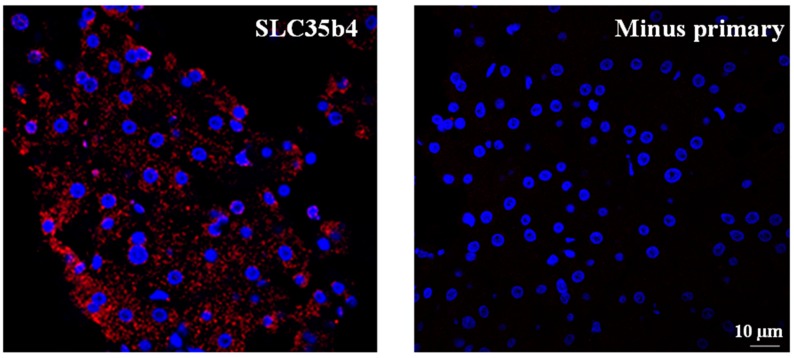
Immunostaining for *SLC35B4* in normal liver tissue. Immunostaining was detected using Alexa fluor555. Cell nuclei were counterstained with Hoechst 33342 dye.

The immunostaining was repeated in primary liver tissues extracted from normal donors to demonstrate the presence of *SLC35B4* (Figure 5). It is important to note that *SLC35B4* protein expression was evident in primary liver cells. The above result gives better relevance to the data previously obtained from tissue culture. However, nutrient response and comparative studies in primary liver cells remain to be elucidated.

### 2.3. SLC35B4 Knockdown Altered the Expression of Multiple Downstream Proteins and Four Were Successfully Identified by MALDI and Mapped to Diabetes-Related Pathways

In the current study, we used 2-DE analysis to look at the liver proteome change in HepG2 cells associated with a decrease in expression of *SLC35B4*. Expression of *SLC35B4* was decreased by around 60% (as assayed by RT-PCR) in HepG2 cells treated with siRNA compared to control cells treated with non-targeting siRNA. 2-DE was performed, and eight differentially expressed protein spots were observed, of which seven were under-expressed with respect to the control and one over-expressed. MALDI-TOF MS/MS analysis was performed in triplicate for each spot. A MASCOT-based MS/MS ion search of raw MS/MS data resulted in a successful identification of four spots: 2633, 3511, 2625, and 3702 (Table 1).

**Figure 6:**
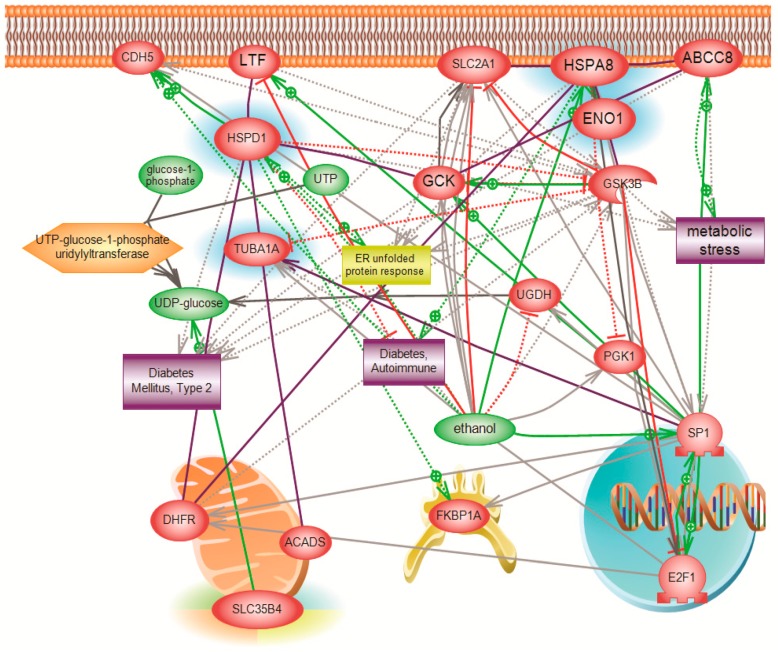
Pathways influenced by identified altered targeted protein. Systems biology analysis of the biological process and molecular function of the four identified genes (HSPD, HSPA8, TUBA1A, and ENO1). The network was generated using the “direct interaction” algorithm to map cellular processes and interactions among altered genes. Of interest, global Pathway analysis revealed an association of these genes to diabetes and to *SLC35B4*. Altered genes are shown in blue color.

The four proteins, HSPD1, HSPA8, TUBA1A, and ENO1, were positively identified with high confidence. The proteins were all under-expressed when *SLC35B4* gene was knocked down. While these proteins are among those identified to be differentially expressed [21], a targeted analysis revealed their involvement in pathways related to diabetes and insulin resistance as well as their link to *SLC35B4*. After a thorough literature search, it was revealed that all four genes identified as downstream targets of *SLC35B4* are involved in the pathogenesis of diabetes. (Figure 6, Table S1).

## 3. Discussion

*SLC35B4* is a novel protein with evidence of involvement in the insulin-glucose homeostasis in the liver. This solute receptor also showed an in vivo impact on predisposing conditions of diabetes (obesity and insulin resistance) and on glucose production [19]. Our report illustrates that *SLC35B4* is present and abundantly expressed in human liver cells. In HepG2 cells, SLC5b4 is localized on the Golgi apparatus and in the ER. Nucleotide sugars are transported into the ER and Golgi, where they become donors of saccharides for the enzymatic reactions of various glycosyltransferases. The properties of several nucleotide sugar transporters, which transport overlapping species of nucleotide sugars, have been studied. Many of these transporters are distributed in the ER and Golgi. Localizing individual nucleotide sugar transporters (NSTs) may be helpful in clarifying their biological role in the glycosylation of macromolecules. Furthermore, the expression of both organelles in HepG2 cells is altered by changing the glucose concentration in the media. These data are important to the mechanisms underlying the functional uptake and production of glucose (sugar nucleotides) by *SLC35B4* in HepG2 cells.

*SLC35B4* is a highly conserved protein that provides a link between the synthesis of nucleotide sugars and the glycosylation process that occurs in the Golgi and ER lumen. Consequently, the regulation of its expression and localization are associated with its function. Since no evidence was reported on PTMs of the *SLC35B4* protein [9], we are encouraged to think that the accumulation of *SLC35B4* in the Golgi apparatus upon glucose stimulation is linked to this protein’s function. This solute receptor is likely to influence the availability of sugar moiety and therefore glycoconjugate (glycoproteins, glycolipids, and proteoglycans) chain synthesis in the Golgi.

**Figure 7:**
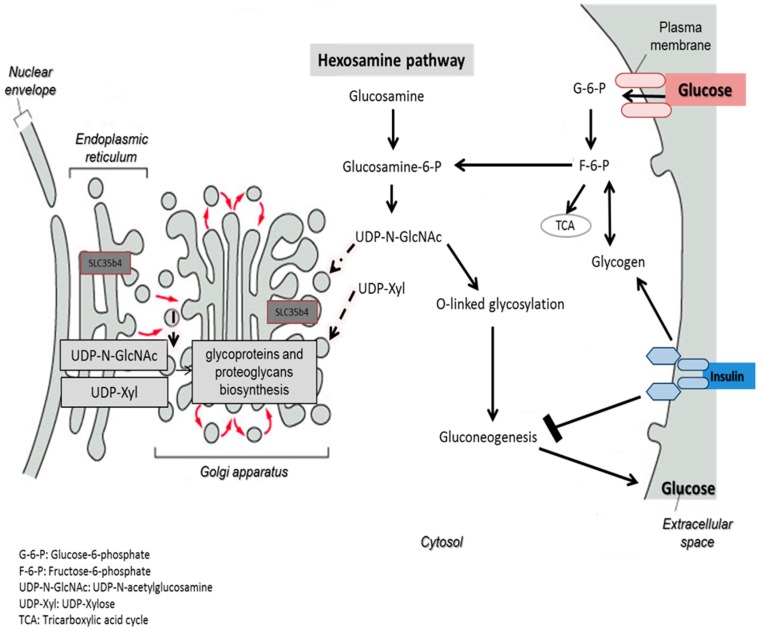
Schematic representation of the potential function of *SLC35B4*. The uptake of UDP-GlcNAc by the *SLC35B4* carrier reduces the O-linked glycosylation by depleting the substrate for the *O*-GlcNAc transferase. This process reduces gluconeogenesis leading to a decrease in glucose production.

One possible hypothesis of the effect on glucose production is that in the presence of exogenous glucose, upregulation of *SLC35B4* decreases the level of UDP-GlcNAc and thereby the O-linked glycosylation in the cytoplasm mediated by OGT. The latter has a direct output on the insulin pathway since it activates gluconeogenesis and glycogenolysis through many signaling intermediates. As a result, *SLC35B4* activation alters the glycosylation pattern inside the cells causing an improvement of the insulin’s ability to inhibit endogenous glucose production. The hypothesis was first reported in Yazbek et al. showing an increase in glucose production in HepG2 cells upon decreasing the expression of *SLC35B4* and is further supported by our localization and expression data (Figure 7).

Furthermore, *SLC35B4* transports two major nucleotides. UDP-xyl is used in three glycosylation processes: (1) protein-*O*-Xylosylation [22] initiating GAG biosynthesis; (2) generation of the Xyl-Xyl-Glc trisaccharide on Epidermal growth factor-like (EGF) repeats of Notch and other proteins; and (3) glycosylation of dystroglycan by the enzyme LARGE [23,24]. The localization of all components of UDP-xyl metabolism in the secretory pathway is not absolutely clear [9,12,15,25]. This makes it uncertain as to where UDP-xyl is exactly produced, transported to, and used. Hence, previous experiments investigating the intra-organelle availability of nucleotide sugars have shown that UDP-xyl and UDP-Glc can also be found in the ER, whereas UDP-GlcNAc, UDP-GlcA, and UDP-Glc can only be found in the Golgi [26]. The identification that the UDP-xyl transporter is in the Golgi apparatus and the ER may begin to decipher the question on functionality.

The function of *SLC35B4* and its involvement in gluconeogenesis was further investigated using a systems biology proteomics approach that involved 2D gel electrophoresis coupled with mass spectrometry. We used differential expression analysis to identify major downstream players affected by the knockdown of *SLC35B4* that can be further investigated. The identified proteins differed between HepG2 liver cells with decreased levels of the receptor as compared to the control. Our stringent inclusion criteria dramatically limited the number of resulting proteins and pathways. However, the biological relevance of the involved proteins was validated by existing published literature linking the identified proteins to diabetes. Thus, inferences could be made on the ability of *SLC35B4* to control expression of the four identified downstream proteins.

One of the major differentially expressed proteins was molecular stress protein HSPD (alternative name, Heat shock protein 60—Hsp60), which is downregulated in *SLC35B4* knockdown tissue by 22-fold. Although a cause-and-effect relationship has not been deciphered, the literature has clearly reported an increase in the levels of Hsp60 in both serum and saliva of diabetic patients types I and II [27]. Beta cell Hsp60 has also been shown to influence T-cell responses and regulate diabetes. The protein is implicated in enhancing the diabetic phenotype through the protective role of epitope p277 on islet function and insulin production in mice and humans. On the contrary, HSPD was implicated in the worsening of diabetes by inducing islet destruction [28,29]. Furthermore, inflammation and stress reactant proteins have long been linked to T2D. Compiling current knowledge suggests that the effect of Hsp60 in T2D could be through its modulatory responses in inflammation, particularly by the activation of toll-like receptors [30]. Knockdown of *SLC35B4* decreased the protein expression of Hsp60, which has been previously reported by our group to lead to an increase in endogenous glucose production. Perhaps more interesting is that *O*-GlcNAcylation of Hsp60 in rat pancreatic β-cells has been shown to increase under hyperglycemic conditions, and it is also implicated in β-islet destruction [31]. Being evidently modified by *O*-GlcNAcylation and the association of this modification with the diabetic phenotype provides a strong implication of the mechanism through which *SLC35B4* could influence the downstream effect on Hsp60.

We also found a significant downregulation in HspA8 (alternative name Hsc70), which has been shown to play a role in protein folding and degradation, protein import into the ER, as well as in trafficking the receptors and vesicles [32]. Increased incidence of diabetes in a transgenic mouse model was attributed to damage in pancreatic beta cells expressing additional cytosolic hsc70 [33]. It was also revealed that HspA8 is upregulated in spleens of Non-obese diabetic mice as compared to controls with decreased incidence of insulitis and diabetes. HspA8 lies within the borders of the Idd2 interval and evidence is suggesting that it is the ideal candidate gene affecting diabetes-related phenotypes within this QTL [34]. Our report further supports the functional link of HspA8 to hyperglycemia.

HSPA8 has been also shown to interact in rat hearts with α-enolase, which is the third protein our report identified as significantly downregulated in the knockdown. Glucose 6-phosphate isomerase and enolase (ENO1) are two important enzymes of glycolysis; they catalyze the interconversions of fructose 6-phosphate to glucose 6-phosphate or of 2-phospho-d-glycerate to phosphoenolpyruvate, respectively. HspA8 deficiency intensified the decrease of α-enolase activity and cell damage in H_2_O_2_-treated H9c2 cells. The protective effect of HspA8 on the cardiomyocytes against oxidative stress is partly associated with its interaction with α-enolase (ENO1) [35]. This same interaction could play a role in the effect of ENO1 on diabetes. ENO1 has been shown to be dysregulated in the gene expression profiles of insulin-sensitive tissues from autopsy donors with or without T2D, particularly upregulated in livers of diabetic individuals [36]. The protein has also presented evidence of sexual dimorphism; it was downregulated in male diabetic rats but upregulated in female diabetic rats by 2-DE-based proteomic analysis [37].

The fourth gene that was significantly downregulated with the knockdown of *SLC35B4* was TUBA1, which represents a major component of microtubules. Microarray analysis in the colorectal cancer SW620 metastatic clone revealed that *O*-GlcNAcase silencing caused *O*-GlcNAcylation elevation and altered the expression of about 1300 genes. TUBA1 was among the category of altered genes that encode proteins responsible for cell–cell interaction and motility [38]. This finding again implicates *SLC35B4* in modulating *O*-GlcNAcylation.

## 4. Materials and Methods

### 4.1. Cell Culture

HepG2 human hepatoma cells were obtained from ATCC (American Tissue Culture Collection, Manassas, VA, USA). These cells are easily maintained and expanded in culture and they have been shown to express a wide range of liver-specific functions. In addition, previous studies using siRNA in HepG2 cells mimicked the effects of the receptor on cellular glucose production. Cells were grown in DMEM high glucose with 4500 mg/L medium supplemented with 10% fetal bovine serum (FBS) (Sigma, St. Louis, MO, USA) and 1% Penicillin-Streptomycin (Sigma, St. Louis, MO, USA). Cells were grown to confluence at 37 °C in a humidified atmosphere containing 5% CO_2_ in air and passaged weekly using 1% trypsin (Gibco, Waltham, MA, USA) to maintain the optimum conditions for exponential growth. Glucose dilution was performed directly in medium from a stock solution of 0.5 M.

### 4.2. Knockdown

A Trilencer-27 siRNA Kit (OriGene, Rockville, MD, USA) was used to knockdown *SLC35B4* in cell culture. Kit information: *SLC35B4* (Human)-3 unique 27mer siRNA duplexes, 2 nmol each (Cat # SR313733) and 2 nmol of nontargeting pool siRNA. Modifications to manufacturer protocol include the use of the DharmaFECT 1 (Dharmacon, Lafayette, CO, USA) transfection reagent and the change of the final siRNA concentration used per well (in a 6-well plate) into 100 nM as described in Yazbek et al. [19].

### 4.3. RNA Extraction

Cells were scraped with Buffer RA1 (Macherey-Nagel, Duren, Germany) in order to maintain RNA integrity. RNA was extracted using Nucleospin RNA II (Macherey-Nagel, Duren, Germany). RNA concentration/purity was measured using Nanodrop ND-1000 (Thermo, Waltham, MA, USA). Five hundred nanograms of extracted RNA were reverse transcribed into cDNA using iScript Reverse Transcription Supermix (Bio-Rad, Hercules, CA, USA).

### 4.4. RT-PCR

RT-PCR was performed using 5X RT-PCR Master Mix (LightCycler Taqman Master, Roche, Basel, Switzerland) in a CFX96 system (Bio-Rad). The reaction was performed as follows: pre-cycle of 50 °C for 2 min then 95 °C for 10 min followed by 50 cycles each consisting of an initial 95 °C step for 10 s, then 57 °C for 45 s and finally 72 °C for 5 s. Gene expression levels were calculated relative to the G6PD control using the 2^−ΔΔCt^ calculation. Primer sequence for SLC34B4 gAACTgCATggTCATAAATATCAgAAgCC: FL and LC640-AgAAgACgAAACCCggAAgTggA: PH.

### 4.5. Protein Extraction

Cells were scraped in 1 mL 2D sample buffer (Bio-Rad) and proteins extracted using the ReadyPrep Total Protein Extraction Kit (Bio-Rad) according to the manufacturer’s instructions.

### 4.6. Protein Quantification

Proteins were quantified via the RC DC protein quantification assay (Bio-Rad) using a Bovine Serum Albumin (BSA) (Bio-Rad) standard curve in 2D sample buffer (Bio-Rad) following the manufacturer’s instructions.

### 4.7. Protein Cleanup

Proteins were cleaned using the ReadyPrep 2D cleanup kit (Bio-Rad) following the manufacturer’s instructions.

### 4.8. 2D-GE

Three biological replicates for each of the control and the knockdown were compared using 2D-GE. Three technical replicates were performed for of each sample. From each of the samples mentioned earlier, 400 μg of cleaned protein solubilized in 300 μL of 2D sample buffer were placed in a 17 cm tray. The 17 cm, pH 3–10 ReadyStrip IPG strips were then covered with 3 mL mineral oil (Bio-Rad). The strips were left to rehydrate overnight. Subsequently, isoelectric focusing (IEF) was performed on a PROTEAN^®^ i12™ IEF System (Bio-Rad) using the following running conditions: (i) Start voltage 0 V; (ii) End voltage 10,000 V; (iii) 40,000 V-h; (iv) Ramp rapid; (v) temperature 20 °C.

SDS-Polyacrylamide gel electrophoresis (SDS-PAGE). After focusing, the strips were then placed on an orbital shaker for 10 min in equilibration buffer I (6 M Urea, 0.375 M Tris-HCl (pH 8.8), 2% *w*/*v* SDS, 20% *v*/*v* glycerol, and 2% *w*/*v* DTT) and for another 10 min in equilibration buffer II (6 M Urea, 0.375 M Tris-HCl (pH 8.8), 2% *w*/*v* SDS, 20% *v*/*v* glycerol, and 2.5% *w*/*v* Iodoacetamide). The strips were cleaned using 1× TGS buffer before being mounted on a 10% (Acrylamide) SDS-PAGE and embedded in an overlay of agarose. The Strips were run with 2D control Precision Plus Protein Std plugs (Bio-Rad). Run conditions were set as 16 mA/gel for 30 min and 24 mA/gel for 5 h.

Staining. After electrophoresis, the gels were washed three times with distilled water and incubated with BioSafe Coomassie stain (Bio-Rad) for 60 min. The gels were washed twice for 15 min each with distilled water.

Imaging. The gel was scanned using Bio-Rad’s GS-800 calibrated imaging densitometer and an image was acquired using the Quantity One software (Bio-Rad). Analysis of differentially expressed proteins was performed using PDQuest software (Bio-Rad). Since multiple studies estimate the total acceptable variation to be around 20% and to be more due to differences from biological variation rather than being just technical, it was important for us to provide three biological replicates as well as three technical replicates per biological sample. Three biological replicates were done for each group, Knockdown and Control. Each biological replicate was performed in triplicate (three technical replicates). This amounts to nine gels per group. To be conservative and minimize false positives and false negatives due to the above-mentioned variation, the selection criteria for differential expression analysis selected exclusively the proteins that were differentially expressed in all of the three technical replicates for each biological sample or control (over-expressed or under-expressed) and among the three biological replicates (in all nine gels). The confidence interval was chosen to be 90% with more than a twofold difference in differential expression, a threshold above the known experimental variation.

### 4.9. In-Gel Tryptic Digestion

After analysis, gel spots of proteins that were differentially expressed were cut and stored. In-gel digestion of the gel spots was performed according to a modified protocol adopted from the University of California, San Francisco Mass Spectrometric Facility. Gel pieces were diced, washed three times with 100 μL of 25 mM NH_4_HCO_3_/50% ACN, and vortexed and dried in a Speed-vac for 20 min. Reduction was attained with the addition of 25 µL of 10 mM DTT in 25 mM NH_4_HCO_3_ and incubation for 1 h at 60 °C. Supernatant was replaced with 25 µL of 55 mM iodoacetamide in 25 mM NH_4_HCO_3_. Alkylation proceeded for 45 min in the dark at room temperature. After discarding the supernatant, gel pieces were washed with 100 μL of 25 mM NH_4_HCO_3_ and dehydrated with 100 μL of 25 mM NH_4_HCO_3_/50% ACN. The gel pieces were dried in a Speed-vac for 20 min, then rehydrated in a digestion buffer containing 25 mM NH_4_HCO_3_ and 12.5 ng/μL TCPK-treated trypsin (Sigma-Aldrich). Tryptic digestion was accomplished by incubating the gel pieces at 37 °C overnight. The digest solutions were transferred to clean 1.5 mL siliconized Eppendorf tubes. Peptides were extracted by three changes of 30 μL 50% ACN/5% formic acid. Every extraction cycle consisted of a vortex step of 20–30 min and a spin step (21,130 g for 2 min). Extracted peptides were added to the digest solutions. The volumes of the extracted digests were reduced to 30 µL using Speed-vac. C18 ZipTips (Millipore, Burlington MA, USA) were used for sample cleanup and concentration to 5 µL according to the manufacturer’s instructions. In-gel digestion for Spot samples 1, 2, 3, and 4 was performed twice and duplicates were mixed in an attempt to achieve a higher concentration, whereas in-gel digestion for Spot samples 5, 6, 7, and 8 was performed once.

### 4.10. Mass Spectrometry

MALDI-TOF MS Plate Preparation: 1 μL aliquots of the in-gel digested protein samples were spotted onto a stainless steel target plate (Opti-TOF TM 384 Well Insert, 123 × 81 mm RevA, Applied Biosystems, Foster City, CA, USA) and overlaid with 1 μL of CHCA matrix solution. Sample spots were air dried before and after matrix spotting. Triplicates of each sample were spotted.

MALDI-TOF MS Calibration: MALDI-MS analysis was performed in reflector-positive mode using a 4800 MALDI TOF/TOF Analyzer instrument operated by the 4000 Series Explorer software version 3.5.1 (Applied Biosystems, USA). The instrument was externally calibrated using 4700 Proteomic Analyzer Calibration Mixture (p/n: 4333604, Applied Biosystems).

MALDI-TOF MS Analysis: MALDI-TOF MS analysis was executed in reflector-positive mode at a laser intensity of 5000 within a mass range of 400 to 4000 Da and a focus mass of 1200 Da. The acceleration voltage and the IS2 voltage were maintained at 20 kV and 16.1 kV, respectively. The extraction delay time was set to 300 ns, while the laser frequency was set to 500 MHz. The minimum signal-to-noise ratio was set to 10 for peak detection. For each sample spot, 800 measurements were accumulated into one spectrum.

MALDI-TOF MS/MS Analysis: an interpretation method was utilized to acquire 10 MS/MS spectra in batch mode for each sample spot. An inclusion list was created for each sample spot based on MALDI-TOF MS spectra obtained in reflector mode. An exclusion list for the CHCA matrix, trypsin, and keratin was created in addition to an adduct exclusion for sodium and potassium at 21.982 and 37.956 *m*/*z*, respectively. MS/MS Spectra were acquired in positive mode using Collision-induced dissociation CID by air (medium dissociation mode). For each sample spot, 1000 measurements were accumulated into one spectrum with the minimum signal-to-noise ratio set to 3.

### 4.11. Data Analysis

MS/MS Ion Search: raw MS/MS spectra were submitted in Mascot Generic Format and searched against SwissProt and NCBInr databases using a licensed MASCOT software version 2.2.04 (matrix science, Boston, MA, USA). Taxonomy was limited to Homo sapiens and a maximum of one missed cleavage was permitted for tryptic digestion. Carbamidomethylation of cysteine residues and oxidation of methionine residues were set as fixed and variable modifications, respectively. MALDI-TOF-TOF was chosen as instrument; consequently, the peptide charge was set to “1+”. The peptide tolerance ranged from 25 ppm to 15 ppm/0.2 Da and fragment tolerance ranged from 0.3 Da to 0.1 Da.

### 4.12. Protein Pathway Analysis

For protein interaction and pathway analysis, Elsevier’s Pathway Studio version 10.0 (Ariadne Genomics/Elsevier) was used to deduce relationships among differentially expressed proteomics protein candidates using the Ariadne ResNetdatabase. The “Subnetwork Enrichment Analysis” (SNEA) algorithm was selected to extract statistically significant altered biological and functional pathways pertaining to each identified set of protein hits. SNEA utilizes Fisher’s statistical test, which is used to determine if there are nonrandom associations between two categorical variables organized by a specific relationship. SNEA starts by creating a central “seed” from all of the relevant entities in the database and retrieving associated entities based on their relationship with the seed (that is, binding partners, expression targets, protein modification targets, and regulation). The algorithm compares the sub-network distribution to the background distribution using a one-sided Mann–Whitney U-Test and calculates a *p*-value indicating the statistical significance of the difference between two distributions. In our analysis, GenBank ID and gene symbols from each set were imported to the software to form an experimental data set. For the reconstruction of networks of pathways, biological processes and Molecular function were evaluated for each single protein hit and its associated targets (networks and pathways).

### 4.13. Immunofluorescence Staining

Liver sections were obtained from anonymous, clinical left over paraffin-embedded tissues. All patients’ identifiers were kept confidential and scientists had no access to any personal or clinical information related to the specimen. Hence, the study does not fall under “human research” and is exempt from Institutional Review Board (IRB) approval. The study protocol conformed to the guidelines of and was given a “Non-Human Research” determination by the ethics committee of the American University of Beirut-IRB. Formalin-fixed, paraffin-embedded tissue blocks were cut into 4-μm-thick sections, and the sections were mounted on glass slides. After deparaffinization in xylene and graded ethanol, the slides were immersed in distilled water. Cultured cells on glass coverslips were fixed in 4% paraformaldehyde solution for 15 min. Cells were then permeabilized with saponin for 15 min.

On both liver tissue and HepG2 cells, a blocking step was performed for 1 h at room temperature with 1% BSA (Sigma, USA). After incubation overnight at 4 °C with primary antibodies against *SLC35B4* (Abcam, Cambridge, UK) or/and GM130 (BD bioscience pharmingen, San Jose, CA, USA) or/and GRP78Bip (Abcam, UK), slides were washed in PBS. The antibodies were diluted in PBS, 10 mg/mL BSA, and samples were placed in a humidified chamber. The rabbit polyclonal antibody *SLC35B4* (0.25 mg/mL), the mouse monoclonal antibody GRP78Bip (1 mg/mL), and the mouse monoclonal antibody GM130 (0.25 mg/mL) were used. Cells were next incubated with the secondary antibody Alexa Fluor 555 of goat anti-rabbit IgG antibody (0.5 mg/mL, Invitrogen, Carlsbad, CA, USA) or Alexa Fluor 647 of goat anti-mouse IgG antibody (0.5 mg/mL; Invitrogen). Cellular DNA was stained by addition of Hoechst 33342 (Sigma, USA) dye at 0.5 mg/mL. Cells incubated without a first antibody were included as controls. After washing, the coverslips were mounted on slides with an anti-fade reagent. Slides were examined using an LSM710 laser scanning confocal microscope (Carl Zeiss, Oberkochen, Germany). The imaging planes for the “ER” and “Golgi” panels were selected based on the signal intensity coming from the antibodies selected for both the Golgi marker (GM130) and ER marker (GRP78).

Immunofluorescence, in situ proximity ligation assays (Duo-link), and confocal microscopy: HepG2 cells were seeded on glass coverslips and then fixed with methanol at −20 °C. Protein–protein interactions were visualized using the Duo-link in situ proximity ligation assay (PLA) system (Olink, Bioscience). Assays were performed using anti-*SLC35B4* (ab107750) and anti-GRP78 (C-terminal ab151269) or anti-GM130 (ab139860) primary antibodies following the manufacturer’s instructions. Images were acquired by confocal microscopy using the LSM710 confocal laser confocal microscope (Carl Zeiss, Oberkochen, Germany).

### 4.14. Western Blotting

Proteins were quantified using a Bradford assay (Bio-Rad, USA). Whole-cell lysates (100 μg) were separated by SDS-PAGE (10%), transferred to PVDF membranes (Bio-Rad, USA), and probed with anti-*SLC35B4* (1 µg/mL, Abcam, UK) followed by horseradish peroxidase-conjugated anti-rabbit IgG (1 µg/mL, Santa-Cruz, Dallas, TX, USA). β-Actin (1 µg/mL, Sigma, USA) was used to ensure equal protein loading. Immune complexes were detected using ECL chemoluminescent system bands and normalized according to the internal control. Bands were quantified using ImageJ software. Measurements were compared using an unpaired Student *t*-test with Welch’s correction. The band appearing at 28 KDa was analyzed as predicted by the product data sheet. A purified *SLC35B4* protein was unavailable to be used as a positive control.

### 4.15. Statistical Analysis

Results were expressed as mean  ±  SEM. All data was analyzed with ANOVA or Student’s *t*-test with multiple testing correction. Two-tailed *p* values less than 0.05 were considered statistically significant.

## 5. Conclusions

Taken together, the findings of this study investigated the downstream effect of *SLC35B4* and its ability to respond to glucose. In the present study, based on the downstream proteomics analysis in human liver cells and the localization assessment of the receptor and the response to glucose, the results obtained demonstrated that the expression of *SLC35B4* is abundant in primary liver cells and can be altered by glucose in a dose-dependent manner in liver cell cultures. In addition, *SLC35B4* is localized in the Golgi apparatus and ER, which has multiple functional implications. The research also identified key players in the downstream function of *SLC35B4*, which could be major downstream modulators of its effect on gluconeogenesis (HspD1, HspA8, TUBA1A, and ENO1). We previously hypothesized that the uptake of UDP-GlcNAc by an *SLC35B4* carrier may have a negative feedback on gluconeogenesis, leading to a decrease in glucose production (Figure 7). Moreover, the finding that some of the downstream proteins are strongly modified by *O*-GlcNAcylation presents evidence to support this hypothesis. The established correlations between *SLC35B4*, the downstream proteins, and their corresponding pathways can be further examined in future studies to provide the mechanism underlying the role of this receptor in glucose production and T2D.

## Figures and Tables

**Table 1:** List of differentially expressed proteins. The means of the expression of the three technical replicates are given for each of the three biological replicates. Controls (C1b, C2b, C3b) versus Knockdown (Kd1b, Kd2b, Kd3b).

Spot Number	Protein Name	Fold Change	C1b	C2b	C3B	Kd 1b	Kd 2B	Kd 3B
3702	ENO1	4.4	103,672.3	248,429.5	185,257.8	50,174.21	39,100.1	33,137.4
2625	TUBA1A	22	1,333,975.0	111,991.6	137,179.6		14,683.9	31,795.8
2633	HSPD1	15	52,867.3	53,057.4	55,377.5	6765.7	1370.8	2595.0
3511	HSPA8	21	41,834.5	10,538.5	46,047.9		1100.2	1963.7

## Supplementary Materials

The following are available online, Table S1: Network Interactions and Pathway Analysis.
